# A Large Pleural Solitary Fibrous Tumor Successfully Resected Using Uniportal Video-Assisted Thoracoscopic Surgery

**DOI:** 10.7759/cureus.42628

**Published:** 2023-07-28

**Authors:** Samah Abu Ajamia, Maram Albandak, Mohammed Ayyad, Yousef Abu Asbeh

**Affiliations:** 1 Internal Medicine, Al-Quds University, Jerusalem, PSE; 2 General Surgery, Al-Quds University, Jerusalem, PSE; 3 Thoracic Surgery Unit, Al-Ahli Hospital, Hebron, PSE

**Keywords:** cardiothoracic surgery, primary pleural tumors, vats, uniportal video-assisted thoracoscopic surgery, solitary fibrous tumor

## Abstract

Solitary fibrous tumors (SFTs) are rare neoplasms arising from submesothelial connective tissue. Typically affecting elderly individuals, SFTs can exhibit malignant characteristics despite most cases being benign. Diagnosis often occurs incidentally on routine chest radiographs, and patients are usually asymptomatic unless the tumor causes compression of adjacent structures. While imaging studies aid in identification, confirmation of the diagnosis requires bronchoscopy with tissue sampling and immunohistochemistry. Surgical excision remains the primary treatment for SFTs, with complete resection being associated with a better prognosis. Our case highlights the successful management of a massive SFT using uniportal video-assisted thoracoscopic surgery (VATS). Regular chest computed tomography (CT) follow-up is important for monitoring SFTs and ensuring timely intervention when necessary.

We present the case of a 54-year-old female with a massive SFT presenting as a pleural tumor in the right lower lobe. The patient was initially asymptomatic, and the diagnosis was made incidentally during routine chest CT follow-up. Uniportal VATS was successfully performed for the excision of the tumor measuring 10x9x6 cm. Our case highlights the successful application of uniportal VATS for the thoracoscopic removal of a huge pleural solitary fibrous tumor.

## Introduction

Primary pleural tumors are rare neoplasms that can be divided into two main categories: diffuse and solitary tumors [1]. They account for 3.7% of all mesenchymal tumors and soft tissue sarcomas, with a reported incidence rate of <1 case/ per million people/ year [2]. The diffuse type is more common and typically presents with a large gross appearance, has a rapidly fatal course, and is strongly associated with asbestos exposure [1]. Solitary fibrous tumors (SFTs), on the other hand, are uncommon neoplasms that are not associated with asbestos and arise from the visceral pleural surface [3,4]. They are also known as localized mesotheliomas and can be benign or malignant, with the prognosis being largely dependent on the stage of the neoplasm. While SFTs are primarily found in the pleura, there have been numerous reports of involvement in extra-pleural sites such as the mediastinum, liver, pancreas, kidney, retroperitoneal spaces, urinary bladder, and extremities [3].

SFTs typically manifest in individuals between the ages of 50 and 60, with no significant gender differences in incidence and age distribution [3]. Although they are frequently asymptomatic, individuals with SFTs may experience nonspecific symptoms such as dry cough, dyspnea, chest pain, or the sensation of a mobile mass in the chest. While SFTs are usually identified incidentally on chest imaging, bronchoscopy with tissue biopsy is typically required for a definitive diagnosis. Surgical removal is the primary treatment for this condition and is often curative; however, in some cases, adjuvant radiotherapy and/or chemotherapy may be necessary [1]. This minimally invasive surgical technique has shown promising results in other thoracic procedures, but its application in the resection of large benign SFTs has not been widely explored.

In this report, we present the case of a 54-year-old patient who was incidentally found to have a large benign neoplasm arising from the pleural surface on a chest computed tomography (CT) scan during breast cancer surveillance. The patient was closely monitored over time, and the tumor was eventually excised due to rapid growth. Histopathological analysis confirmed the diagnosis of a benign solitary fibrous tumor, which was managed successfully with uniportal video-assisted thoracoscopic surgery (VATS) excision.

## Case presentation

A 54-year-old patient was diagnosed with breast cancer in 2013 and was referred to the surgical ward for a left breast lumpectomy. At that time, the patient had an incidental finding of a localized swelling in the left breast without nipple discharge, lymphadenopathy, or noticeable changes in the skin. Mammography and breast ultrasound (U/S) were performed and confirmed findings consistent with a malignant lesion in the left breast. Fine needle aspiration (FNA) and tru-cut biopsy revealed moderately differentiated invasive ductal mammary carcinoma. Subsequently, a whole-body CT scan was performed to check for metastatic disease, which was unremarkable except for an incidental right-sided pleural mass measuring 4.8x1.3 cm. A CT-guided biopsy of the pleural mass revealed fibrofatty tissue infiltrated by reactive lymphocytes and histiocytes with extensive fibrosis, suggestive of an SFT. The patient's breast carcinoma was treated with chemotherapy, surgical resection, and biological treatment, while the fibrous tumor was managed with observation and close follow-up.

In 2017, the patient returned to the clinic complaining of right-sided chest pain. A chest x-ray was done and showed an oval-shaped solid mass with well-defined borders in the right lower lung zone. A subsequent CT scan confirmed an increase in the size of the right-sided pleural mass, measuring 6.2x2 cm. At that time, the patient refused surgical intervention and was treated with nonsteroidal anti-inflammatory drugs (NSAIDs) with marked improvement in her pain.

**Figure 1:**
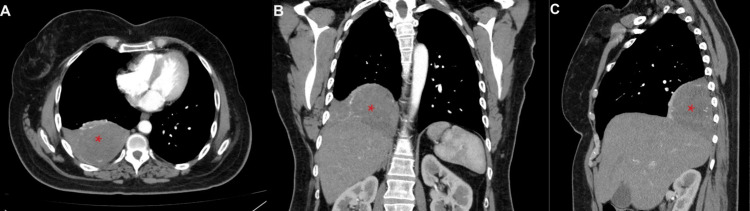
Contrast-enhanced CT scan of the chest revealing a large right-sided pleural solid vascular mass consistent with a solitary fibrous tumor. Combined axial (A), coronal (B), and sagittal (C) views of the CT scan of the chest with contrast. A large right-sided pleural solid vascular mass measuring 10x9x6 cm is visualized (asterisk), along with passive atelectasis in the base of the right lung, left apical fibrotic changes consistent with postradiation fibrosis, and left adrenal thickening.

**Figure 2:**
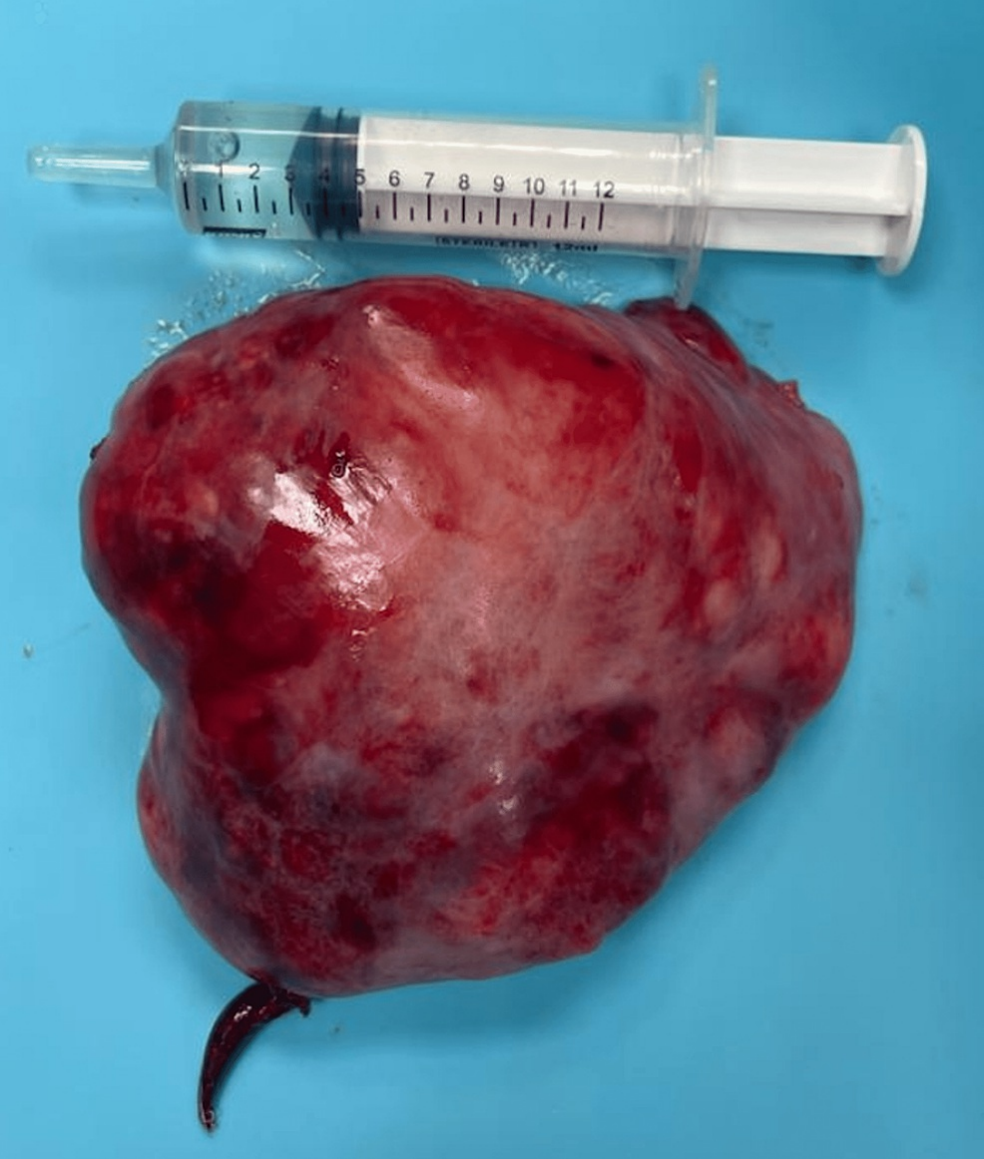
Excised right-sided pleural mass using uniportal VATS measuring 10x9x6 cm. The histopathology evaluation confirmed a benign solitary fibrous tumor. VATS: Video-assisted thoracoscopic surgery

**Figure 3:**
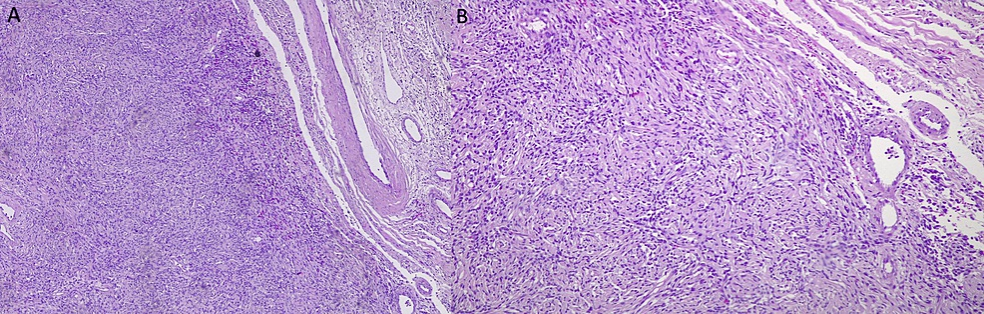
Microscopic examination of the tumor. The histopathology is consistent with a low-grade solitary fibrous tumor of the pleura. (A): Hematoxylin and eosin (H&E) stain, 100x; fairly capsulated tumor of the pleura. (B): H&E stain, 200x; ill-defined short fascicles of ovoid to fusiform spindle cells with indistinct cell borders arranged haphazardly.

**Figure 4:**
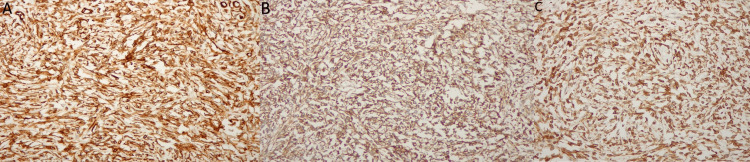
Immunohistochemical evaluation of the pleural mass revealing positive staining for CD34, CD99, and Bcl-2. (A): Immunohistochemistry, CD34: Diffuse positive staining. (B): Immunohistochemistry, CD99: Diffuse positive staining. (C): Immunohistochemistry, BCL2: Diffuse positive staining.

Four years later, the patient's symptoms worsened and became nonresponsive to NSAIDs. A contrast-enhanced CT of the chest revealed an enlargement of the right pleural mass measuring 10x9x6 cm, along with passive atelectasis in the base of the right lung, left lung apical fibrotic changes consistent with postradiation fibrosis, and left adrenal thickening (Figure 1). After consultation with the surgical team, the patient underwent right uniportal video-assisted thoracoscopic surgery, in which a double lumen intubation was applied, with selective lung ventilation and deflation of the lung at the operative side, the patient was positioned in a lateral decubitus position. During the procedure, a sizable tumor was found attached to the visceral pleura of the right lower lobe. To access the tumor, a 4 cm incision was made in the fifth intercostal space. Additionally, mild adhesions were noted, connecting the tumor to both the diaphragm and the chest wall. After complete dissection of the adhesions using an energy device, the tumor was identified as a pedunculated mass attached to the visceral pleura by a small pedicle. Using an EndoGia stapler 45 mm, a medium thick load, and a thoracoscopic endo bag to prevent seeding, the tumor was completely separated from its pleural attachment and excised. Although the extraction of the tumor throughout the uniportal incision was challenging due to its large size, it was eventually removed without complications (Figure 2). The tumor was sent for histopathological and immunohistochemical evaluation, which revealed positive staining for CD34, CD99, and Bcl-2 and negative staining for desmin and S100, confirming the diagnosis of a low-risk SFT (Figures 3, 4).

Postoperatively, the patient was transferred to the surgical intensive care unit (ICU) for observation and was initiated on intravenous analgesics, fluids, and prophylactic antibiotics. The patient was transferred to the surgical ward as her condition continued to improve. Additionally, a chest x-ray was performed and showed no evidence of pneumothorax or pleural effusion; thus, the chest tube was removed. Four days postoperatively, the patient was discharged home with no complications. She was counseled to follow-up at an outpatient surgical clinic two weeks postdischarge. Three months later, she underwent a chest CT scan to assess for tumor recurrence, which was unremarkable.

## Discussion

SFTs are rare neoplasms of the pleura and comprise less than 5% of primary pleural tumors. They originate from the mesenchymal cells of the submesothelial layer of the pleura and have an indolent clinical course. The etiology of SFTs still remains unknown without clearly established risk factors. There is no current evidence linking SFTs to environmental exposures like smoking, asbestos, and radiation [5]. 

SFTs, previously described as “benign mesotheliomas”, equally affect men and women, presenting in the 6th and 7th decades of life with an estimated frequency of 2.8 per 100,000 individuals [6]. While the majority of SFTs of the pleura are benign, 20% of cases are malignant [5]. The neoplasm is usually found incidentally on routine chest radiographs, and patients are typically asymptomatic. Nonetheless, nonspecific respiratory symptoms include cough, shortness of breath, chest pain, and hemoptysis. In addition, around 20% of patients demonstrate digital clubbing or hypertrophic osteoarthropathy, and 2-4% of patients have recurrent hypoglycemic episodes [1]. Symptomatic presentation is more common in larger and malignant tumors. Compression of the inferior vena cava can lead to lower extremity edema, while airway obstruction may cause obstructive pneumonitis in some cases [5]. Patients can rarely present with paraneoplastic syndromes, such as Doege-Potter syndrome and Pierre-Marie Bamberg syndrome [5]. 

SFTs of the pleura are usually found as single masses with a size ranging from 1 to 39 cm and an average weight varying from 100 to 400 mg [1]. The initial diagnostic step involves obtaining imaging like CT and magnetic resonance imaging (MRI). CT will reveal a well-circumscribed homogeneous mass originating from the pleura, as evidenced in our patient, which enhances with contrast administration due to its vascularity. MRI can further differentiate the tumor from the surrounding structures and delineate the extent of its involvement. An SFT is more likely to be malignant if it exhibits features such as large size, pleural effusion, and necrosis. To reach a definitive diagnosis, histologic examination of tissue samples is required. [5]. The dominant feature of these tumors is characterized by an irregular pattern of randomly arranged fibroblast-like spindle cells and connective tissue, often referred to as the "patternless pattern." A prominent network of capillaries and large vessels is observed in 25% of cases [1]. The presence of hypercellularity, a high mitotic index (more than four per high-powered field), necrosis, and hemorrhage suggest malignancy [7]. 

Furthermore, immunohistochemical testing is an invaluable tool for making a definitive diagnosis given the patternless histological pattern of this tumor, which helps distinguish SFTs from mesotheliomas [5,8]. SFTs are defined by the presence of CD34, Bcl-2, CD99, and vimentin, while lacking other markers like desmin, S100 protein, epithelial membrane antigen (EMA), and low-molecular-weight cytokeratins [5]. 

The preferred treatment for SFTs of the pleura is surgical resection with 1-2 cm clear margins by either thoracotomy or VATS [1]. Pedunculated tumors can be resected by VATS, while the recommended treatment approach for larger tumors with a sessile morphology attached to the parietal pleura involves surgical resection with wide margins [5]. Interestingly, despite the large size of our patient’s tumor, it was successfully resected with uniportal VATS excision, showcasing the potential of this technique in the management of SFTs. However, it is crucial to note that the application of uniportal VATS for SFT resection requires a skilled surgeon experienced in this technique and access to the necessary equipment and resources. Continued exploration and evaluation of uniportal VATS in the context of pleural SFTs can contribute to the growing body of evidence supporting its efficacy as a viable surgical option. Further studies and exploration are warranted to validate the safety and efficacy of uniportal VATS in a larger cohort of patients.

Complete surgical removal of the tumor with microscopically clear surgical margins is the best prognostic indicator [1]. The recurrence rate of benign tumors is 5%, while the estimated recurrence rate of malignant tumors is 60%. Recurrent, unresectable, and metastatic tumors exhibit a higher mortality rate [5]. The 10-year overall survival rate for patients who underwent SFT resection with negative margins falls between 54% and 89% [2]. Given the risk of late recurrence, patients with these tumors need to be monitored for an extended period. In a study by Lahon et al., 157 patients underwent complete resection of their SFTs. Fifteen patients (10%) experienced tumor recurrence despite resection with negative margins, with a median time to recurrence of 29 months [9]. As these tumors are rare, no systematic evaluation has been conducted to determine the effectiveness of adjuvant therapy for benign pleural SFTs [8]. 

Fattahi et al. studied 13 patients diagnosed with benign SFTs of the pleura, with a mean age of 50.46 years [8]. Among them, two patients had a history of unrelated malignancies, including germinoma and a lung mass, and one patient had a prior history of SFT of the pleura. Of the 13 patients, 12 underwent complete surgical resection through an open thoracotomy, while one patient was treated with VATS. The sizes of the tumors varied from 2 to 20 cm [8]. In our case, the SFT was incidentally discovered during evaluation for an unrelated breast malignancy and was successfully treated with VATS despite its huge size, with no evidence of recurrence during follow-up. Similarly, Shen et al. reported the successful resection of a giant SFT (18x12x6 cm) with minimally invasive VATS. Thus, endoscopic surgery can be a viable treatment approach for the resection of large SFTs after careful consideration [10]. While surgical resection remains the treatment of choice for SFTs of the pleura, further systematic assessment is necessary to evaluate the efficacy of adjuvant therapies in cases of recurrent and/or malignant SFTs.

## Conclusions

SFTs of the pleura are rare mesenchymal neoplasms characterized by haphazard proliferation of spindle-shaped cells surrounded by fibrous stroma of collagen fibers. Surgical resection with microscopically clear margins either through thoracotomy or VATS is the curative treatment of choice. Our case highlights the successful application of uniportal VATS for the thoracoscopic removal of a huge pleural SFT. Using a minimally invasive approach for the resection of large pleural SFTs is feasible, given the availability of skilled surgeons, adequate experience, and appropriate facilities. Long-term surveillance is recommended due to the possibility of late recurrence. However, further studies are warranted to evaluate the efficacy and outcomes of uniportal VATS in a broader cohort of patients with large pleural SFTs.
